# Olaparib in Epithelial Ovarian Cancer

**Published:** 2016-11-01

**Authors:** Paula J. Anastasia

**Affiliations:** Cedars-Sinai Medical Center, Los Angeles, California

Epithelial ovarian cancer (EOC) is the most fatal of the gynecologic malignancies, due to the lack of early screening and detection modalities and the advanced stage at the time of diagnosis (American Cancer Society [ACS], 2016). The diagnosis of EOC is often interchanged for or includes fallopian tube carcinoma (FTC) and primary peritoneal carcinoma (PPC), even though these are three distinct cancer origins. The frequent occurrence to categorize these three cancers in the EOC statistics is because the treatment and prognosis are the same (National Comprehensive Cancer Network Clinical Practice Guidelines in Oncology [NCCN], 2016a, 2016b). The use of EOC throughout this article will infer inclusion of FTC and PPC respectively.

There are several histology subtypes for EOC, although high-grade serous (HGS) adenocarcinoma is the most common. Treatment of women newly diagnosed with EOC includes surgery and chemotherapy. Most patients will respond to surgical debulking and either neoadjuvant or adjuvant platinum-based chemotherapy. Unfortunately, more than 70% of patients will have a recurrence of their disease.

Treatment options for recurrent disease are primarily determined by the platinum-free interval (PFI) and the side-effect profile from prior therapy (NCCN, 2016b). The PFI is significant because women who recur more than 6 months from their last platinum chemotherapy, also referred to as platinum-sensitive, will often respond to retreatment with a platinum agent. Notwithstanding, the majority of women with EOC will eventually become resistant to platinum-based therapy, although there are other non–platinum treatment options (Ledermann et al., 2014).

Women with hereditary mutations in the *BRCA1* or *BRCA2* (*BRCA1/2*) genes have an increased risk of several cancers, including EOC. Deleterious mutations occur more frequently in patients with platinum-sensitive EOC than platinum-resistant EOC (Ledermann et al., 2014). Mutations in a *BRCA* gene are deficient in the double-strand DNA break-repair process of homologous recombination (HR). Double-strand breaks can be repaired by an error-prone nonhomologous end-joining repair (NHEJ) system, with a risk for chromosomal alterations and cell death, or survival with DNA mutations and cancer susceptibility (Sessa, 2011; Underhill, Toulmonde, & Bonnefoi, 2011; Walsh, 2015). In up to 50% of patients with HGS tumors, the tumor cells may be deficient in HR as a result of germline (hereditary) or somatically (naturally occurring) acquired *BRCA1/2* mutations (Ledermann et al., 2014; Sessa, 2011). Cancer cells with abnormal function of these two genes or other genes implicated in similar DNA-repair pathways to *BRCA1/2* are said to have BRCAness. This is when the patient is born with intact BRCA repair function but acquires a HR deficiency, which interferes with DNA repair (Walsh & Hodeib, 2016).

## INDICATION

Olaparib is approved as a single agent for patients with recurrent advanced EOC who have a deleterious or suspected deleterious germline *BRCA* mutation and have received three or more lines of chemotherapy. There is a US Food and Drug Administration (FDA)-approved laboratory companion kit for BRCA1/2 testing called BRACAnalysis CDx (Myriad Genetics, 2016). However, patients who have had prior BRCA testing are not required to have repeat testing through the companion kit.

## MECHANISM OF ACTION

Poly (adenosine diphosphate [ADP-ribose]) polymerase (PARP) represent a class of 17 enzymes involved in cellular function that interrupt DNA repair, stability, and cell death. These PARPs are involved in base excision repair (BER) or single-strand DNA breaks (Dalton & Coleman, 2015; Underhill et al., 2011). Olaparib is a potent PARP inhibitor that is selectively cytotoxic to cells while preserving repair efficient cells expressing deleterious *BRCA1/2*. It targets or blocks PARP from repairing damaged single-strand DNA breaks (Fong et al., 2010). These PARP proteins survey and correct damaged DNA cells by binding and recognizing DNA damage.

When unrepaired single-strand breaks are replicated, they rely on double-strand breaks to form, which require DNA damage to be repaired or else the cells die. Normal cells have two ways to repair DNA damage: HR deficiency and PARP. Having one or the other repair mechanism intact allows cell viability (Farmer et al., 2005; Walsh & Hodeib, 2016), whereas a deficiency in both repair pathways results in cell death or synthetic lethality. Cells that are BRCA-deficient will either die because of genomic instability or survive with DNA mutation. Thus, a PARP inhibitor takes away cancer cells’ only repair mechanism, resulting in cell death (Underhill et al., 2011; Table 1).

**Table 1:**
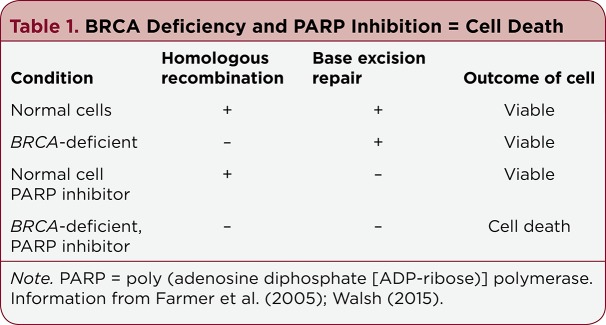
BRCA Deficiency and PARP Inhibition = Cell Death

## METHOD OF ADMINISTRATION

Olaparib has an approved dose of 400 mg twice daily. It is packaged as a 50-mg oral capsule, which translates into dosing 8 capsules in the morning and 8 capsules in the evening. Patients are instructed to take the capsules whole, and not to chew, open, or dissolve. Medication may be taken with or without food. It is metabolized by CYP3A, and therefore use of strong CYP3A inducers (e.g., phenytoin, rifampicin, St. John’s wort, and carbamazepine) and moderate CYP3A inhibitors (e.g., bosentan, efavirenz, etravirine, nafcillin, and modafinil) is prohibited. Patients should be instructed to avoid grapefruit and Seville oranges (AstraZeneca, 2014). Inducers of CYP3A may reduce the potential toxicity of olaparib, whereas CYP3A inhibitors may increase the potential toxicity of olaparib. If inhibitors cannot be avoided, dose reductions to 150 mg orally twice daily is recommended for strong CYP3A inhibitors and 200 mg orally twice daily for moderate inhibitors (AstraZeneca, 2014).

## CLINICAL STUDIES

The indication for olaparib was granted under accelerated approval based on an objective response rate (ORR) and duration of response in a heavily pretreated population of 137 patients with advanced ovarian cancer who had a germline *BRCA* mutation. This is known as study 42 (Kaufman et al., 2015; Tewari, Eskander, & Monk, 2015). The ORR was 34%, with a median duration of response of 7.9 months. The majority of the patients were platinum-resistant or -refractory, and over half of them had five or more lines of chemotherapy (AstraZeneca, 2014). This approval was granted late in 2014 and is contingent upon verification of clinical benefit from studies of olaparib in ovarian cancer (SOLO).

Audeh et al. (2010) provided proof of concept of the efficacy of olaparib in women with recurrent ovarian cancer who had received at least three regimens of chemotherapy. All 57 patients had a deleterious *BRCA1/2*. Patients were randomized to receive either 400 mg or 100 mg orally twice daily. The 400-mg group had an ORR of 33%, compared with 13% in the 100-mg group. The group who received the 400-mg twice daily dose had more nausea, fatigue, and anemia than those in the lower-dose cohort (Table 2).

**Table 2:**
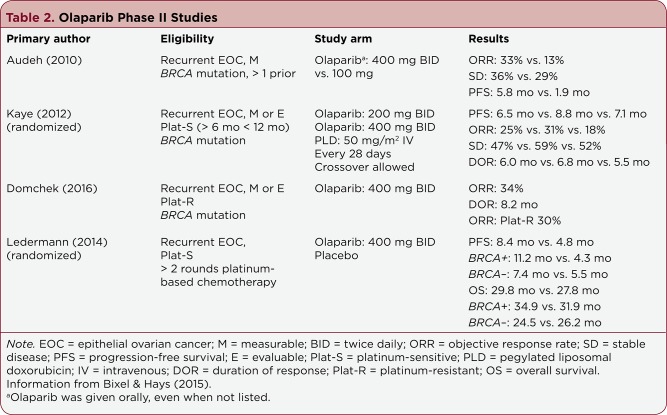
Olaparib Phase II Studies

In a randomized, open-label phase II study (sometimes referred to as study 41), patients with recurrent platinum-sensitive EOC and up to 3 prior regimens were randomized to receive olaparib 200 mg twice daily on days 1 to 10 plus paclitaxel and carboplatin, followed by maintenance olaparib at 400 mg twice daily, vs. chemotherapy alone (Oza et al., 2015). Patients in the combination group had a longer progression-free survival (PFS) of 12.2 months vs. 9.6 months. Patients with a deleterious *BRCA* did better than those without a deleterious mutation. As expected, the combination arm reported more adverse events. The most common grade 3/4 events were neutropenia and anemia (Oza et al., 2015).

A phase II double-blind randomized maintenance study of olaparib at 400 mg twice daily vs. placebo reported a longer PFS of 8.4 months with olaparib compared with 4.8 months with placebo (Ledermann et al., 2014). Further analysis stratified patients for BRCA status, showing those with deleterious *BRCA1/2* had a significant improvement in PFS of 11.2 months compared with 4.3 months. However, overall survival (OS) was similar between patients with germline *BRCA* mutations and those with normal BRCA status (Ledermann et al., 2014). These data were presented for accelerated approval to the Oncologic Drugs Advisory Committee (ODAC), which voted against FDA accelerated approval for olaparib as a maintenance therapy in the United States until there were further data.

The European Commission approved olaparib for the use of platinum-sensitive recurrence *BRCA*-mutated EOC as maintenance therapy after complete or partial response to platinum-based therapy (Walsh & Hodeib, 2016; Tewari et al., 2015).

Olaparib received US FDA accelerated approval on December 19, 2014, in patients with known or suspected deleterious *BRCA1/2* ovarian cancer who have received at least three prior chemotherapy regimens. In a nonrandomized phase II trial by Kaufman et al. (2015), 298 patients with germline deleterious *BRCA1/2* solid tumors, including breast, ovarian, prostate, and pancreatic cancers, received olaparib at 400 mg orally twice daily. The ORR was 26.2%, with a median PFS of 7.9 months. The cohort of 193 patients with EOC who were platinum-resistant had a median of 4.3 prior chemotherapy regimens. The ORR (n = 60) was 31.1%, and 40% had stable disease, with a median PFS and OS of 7 and 16.6 months, respectively (Tewari et al., 2015).

The updated analysis of the phase II study 42 stratified those patients with EOC who had a germline mutation and more than 3 regimens of chemotherapy. In patients with a germline *BRCA1/2* mutation, 80% had received 3 or more lines of chemotherapy and also had measurable disease. The ORR was 34%; for patients who were platinum-sensitive, the ORR was 46%, and for those who were platinum-resistant, the ORR was 30% (Domchek et al., 2016).

Ongoing phase III studies are in progress to determine whether olaparib will maintain its current accelerated indication or additional indications will be granted, such as its use in maintenance therapy. SOLO 3 is a phase III randomized trial of olaparib vs. physicians’ choice of four chemotherapy options in patients with relapsed platinum-sensitive ovarian cancer who have had two or more lines of platinum-based chemotherapy. SOLO 2 is a phase III randomized, placebo-controlled chemotherapy trial of maintenance olaparib vs. placebo following second-line complete or partial response in platinum-sensitive patients (Tewari et al., 2015; Walsh & Hodeib, 2016).

## ADVERSE EVENTS

Patients with recurrent EOC and a deleterious *BRCA1/2* gene who received at least 3 lines of chemotherapy were enrolled in monotherapy with oral olaparib (400 mg twice daily). Domchek and colleagues (2016) summarized the most common side effects from study 42, including only the 193 patients with ovarian cancer who received olaparib at 400 mg orally twice a day (Table 3). The most common side effects of olaparib for all grades were fatigue, nausea and abdominal pain or discomfort, and anemia (AstraZeneca, 2014). A total of 20% of patients had grade 3/4 anemia. Six patients died due to adverse events, including acute myeloid leukemia (AML), chronic obstructive pulmonary disease, cerebrovascular accident, wound dehiscence, and pulmonary embolism. Pneumonitis occurred in less than 1% of patients treated with olaparib; although it is rare, it can be fatal, so patients should be evaluated for signs and symptoms of respiratory changes, fever, dyspnea, and chest radiology abnormalities. Olaparib should be discontinued if pneumonitis is suspected (AstraZeneca, 2014).

**Table 3:**
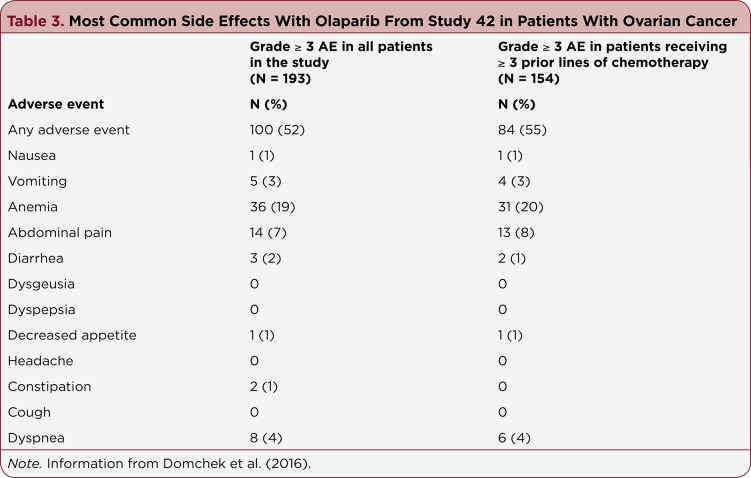
Most Common Side Effects With Olaparib From Study 42 in Patients With Ovarian Cancer

Myelodysplastic syndrome (MDS) and AML have been reported in 6 of 298 patients (2%) and in 3 of 126 randomized placebo-controlled trials. It is recommended that before patients begin olaparib, they have recovered from previous chemotherapy hematologic toxicities. For patients who have prolonged grades 2–4 hematologic events, especially thrombocytopenia, it is recommended to hold olaparib and monitor blood cell counts weekly until recovery to grade 1. If the blood cell levels do not recover within 4 weeks, the patient should be referred to a hematologist for further evaluation for MDS or AML (AstraZeneca, 2014).

## ROLE OF THE ADVANCED PRACTITIONER

The NCCN (2016a, 2016b) has included olaparib in its guidelines as a fourth-line treatment for women with EOC and who carry a deleterious *BRCA1/2* gene. Prior to prescribing olaparib, advanced practitioners should be familiar with the patients’ prior chemotherapy tolerance, side effects, and residual effects. Patients’ medications should be reviewed to assess for any potential drug interactions with known CYP3A4 inhibitors or inducers. As with any cytotoxic therapy, the general rule is to allow a 4-week washout before starting a new treatment, although individualized evaluation is essential.

With any new targeted therapy, there is excitement and hope for patient response; however, patients need to be vetted for oral adherence, as the dosing includes 16 pills a day. Patients should be instructed on the most common side effects of fatigue, nausea, and hematologic side effects. Although the package insert recommends monthly monitoring of blood cell counts, patients who have a history of chemotherapy-induced hematologic events may be considered for weekly monitoring of complete blood cell counts for the first month.

Patient education should include informing patients about the risk for a secondary malignancy such as AML or MDS, including pertinent blood cell count criteria, in which a referral to a hematologist should be made. In situations when a patient has a grade 2–4 hematologic toxicity, the dose of olaparib should be reduced or held, respectively. For patients with mild renal impairment, there is a 1.5-fold risk in mean exposure, so a comprehensive metabolic panel should be obtained at monthly evaluations, with a focus on elevated serum creatinine levels. The effect of hepatic impairment with olaparib use has not been studied (AstraZeneca, 2014).

Olaparib can be ordered through the Biologics specialty pharmacy found on its website (https://www.lynparzahcp.com [AstraZeneca, 2014]) by a physician, physician assistant, or nurse practitioner. Medication order forms can be downloaded and faxed with the patient’s chemotherapy history to Biologics. Biologics also employs nurses to contact the patient bimonthly and report ongoing side effects to the prescribing practitioner. Hospitals may contract with distributors for possible medication dispensing.

## SUMMARY

Advanced ovarian cancer is a chronic disease, with most patients receiving multiple lines of chemotherapy. Olaparib is a targeted agent for a select population of women with at least three prior lines of chemotherapy and a deleterious *BRCA1/2*. The patient population eligible for olaparb will already be heavily pretreated, with at least three prior lines of chemotherapy.

Baseline assessment for potential risk of adverse events will be essential for the advanced practitioner. Future studies may show that olaparib may also benefit women with a somatic *BRCA1/2* mutation or other HR deficiency pathways such as RAD51C/D or BRIP1 (NCCN, 2016a, 2016b). The current indication of olaparib may change, based on results from the SOLO studies. Additionally, there may be future indications for both men and women who have deleterious *BRCA1/2*-associated cancers such as breast, prostate, pancreatic, and melanoma, as more study results are reported.
